# Small intestinal metastasis of triple-negative breast cancer mimicking lymphoma: imaging findings and case report

**DOI:** 10.3389/fonc.2026.1900018

**Published:** 2026-07-09

**Authors:** Lin-Ying Ma, Xiao-Shan Huang, Shu-Feng Fan, Li-Ming Xue

**Affiliations:** Department of Radiology, The Second Affiliated Hospital of Zhejiang Chinese Medical University, Hangzhou, China

**Keywords:** anemia, breast cancer, computed tomography, gastrointestinal metastasis, small intestinal metastasis

## Abstract

Small intestinal metastasis from breast cancer is a rare entity with non-specific clinical presentations, rendering it susceptible to missed or delayed diagnosis. Herein, we present a rare case of breast cancer metastasizing to the small bowel, with unexplained anemia as the sole initial clinical complaint.The patient manifested persistent dizziness and palpitations, accompanied by repeatedly positive fecal occult blood testing, and carried a well-documented history of surgical resection for bilateral breast malignancy.Contrast-enhanced computed tomography (CT) of the small bowel demonstrated segmental, asymmetric wall thickening with adjacent lymphadenopathy and no luminal stenosis; the thickened wall exhibited heterogeneous enhancement, raising initial suspicion of lymphoma. Subsequent double-balloon enteroscopy(DBE) identified a large circumferential deep ulcerative lesion, which was endoscopically indistinguishable from primary small bowel adenocarcinoma or intestinal lymphoma. The definitive diagnosis of small intestinal metastasis from breast cancer was established by histopathological and immunohistochemical evaluation, in conjunction with the patient’s oncological history. A review of the literature indicates that small intestinal metastasis from breast cancer is more commonly associated with invasive lobular carcinoma, and affected patients usually present during routine surveillance or with abdominal pain. In the present case, the primary pathology was invasive ductal carcinoma of triple-negative phenotype (TNBC), and the patient’s initial presentation with anaemia constitutes an atypical manifestation. Clinicians should maintain a high index of suspicion for small bowel metastasis in breast cancer survivors who develop unexplained anemia, positive fecal occult blood, or abnormal small intestinal imaging, even in the absence of classic gastrointestinal symptoms. A multidisciplinary approach integrating imaging, endoscopy, pathology, and immunohistochemistry is essential to minimise diagnostic delay and avoid misdiagnosis.

## Case report

A 70-year-old female was admitted to our hospital with a 4-month history of dizziness, fatigue and palpitations, which had worsened over the preceding 5 days. The symptoms had begun 4 months earlier without identifiable precipitating factors; they were exacerbated by physical activity and relieved by rest, and were not accompanied by abdominal pain, distension, nausea, or vomiting. Faecal occult blood testing (FOBT) was positive (+++) on two separate occasions (30 October and 21 December 2025) at a community clinic. Subsequently, she presented to our outpatient department, where a haemoglobin level of 57 g/L was documented. She was therefore admitted with a working diagnosis of severe anaemia and gastrointestinal bleeding of undetermined origin.

## Medical history

The patient had a history of bilateral breast cancer. In March 2022, she underwent right radical mastectomy. Pathological examination revealed invasive ductal carcinoma of the right breast with squamous differentiation. Immunohistochemical staining yielded the following results: HER2 (0), Ki-67 (30%+), oestrogen receptor (ER) negative, progesterone receptor (PR) negative, androgen receptor (AR) strongly positive (85%), CK5/6 (partial positive), E-cadherin (membranous positive), GATA-3 (positive), and P63 (partial positive). On 14 April 2023, she underwent left total mastectomy with left axillary lymph node dissection for left breast cancer and ipsilateral axillary lymphadenopathy. In April 2024, a left chest wall mass was identified, and biopsy revealed infiltrating adenocarcinoma consistent with a breast primary on immunohistochemical analysis. From July to November 2024, the patient received a clinical trial regimen comprising targeted therapy, immunotherapy, and combination chemotherapy, administered as follows: lenvatinib mesylate 8 mg orally once daily; sintilimab 200 mg intravenously on day 1 of each cycle; and nab-paclitaxel 125 mg/m² intravenously on days 1 and 8 of each cycle. After completing the combined phase, sintilimab monotherapy was continued as maintenance therapy until July 2025, when it was discontinued because of drug intolerance and myelosuppression.

On admission, physical examination showed pallor consistent with anemia, and no remarkable abdominal signs. Laboratory tests revealed white blood cell count 1.9×10^9^/L (reference range: 3.5–9.5×10^9^/L), hemoglobin 57 g/L (reference range: 115–150 g/L), mean corpuscular volume 70.4 fL (reference range: 82–100 fL), and ferritin 9.2 ng/mL. Serum folate was >20 ng/mL (elevated), while serum vitamin B12 was 587 pg/mL (reference range: 197–771 pg/mL, within normal limits). Red blood cell parameters were as follows: red blood cell (RBC) count 2.81×10¹²/L (reference range: 3.8–5.2×10¹²/L, decreased), hematocrit (HCT) 0.20 L/L, mean corpuscular hemoglobin (MCH) 20.3 pg, mean corpuscular hemoglobin concentration (MCHC) 288 g/L (reference range: 316–354 g/L, decreased), and red cell distribution width (RDW) 22.2% (reference range: 11%–16%, elevated). Reticulocyte (RET) fraction was 3.45% (reference range: 0.5%–1.5%, elevated), and absolute reticulocyte count was 97×10^9^/L (reference range: 24–98×10^9^/L, mildly elevated).Following admission, non-contrast abdominal CT revealed suspected thickening of the small bowel wall. Contrast-enhanced CT of the small bowel demonstrated segmental, localised, asymmetric circumferential wall thickening involving the third and fourth groups (corresponding to the jejunum and portions of the ileum). The thickened bowel wall demonstrated heterogeneous enhancement, with no luminal stenosis. Multiple enlarged lymph nodes were observed in the perienteric region. Lymphoma was initially considered the leading diagnostic consideration (**Figures 1A–C**). Subsequent DBE revealed a deep ulcerative lesion in a segment of the fourth group of the small bowel, with a circumferential extent of approximately 5–6 cm. The lesion was covered with yellowish-white exudate, exhibited a firm consistency, and was surrounded by mucosal oedema and erosions, with associated local luminal stenosis. Multiple biopsy specimens were obtained from the lesion. The lesion was friable and prone to bleeding after biopsy. Endoscopic findings were suggestive of a small intestinal ulcerative lesion, with small intestinal carcinoma or lymphoma among the differential diagnoses (**Figure 2**). Histopathological examination of the biopsy specimens revealed poorly differentiated adenocarcinoma with necrosis in specimen 1, and chronic mucosal inflammation in specimen 2. Immunohistochemical staining demonstrated the following profile: GATA-3 (+), CK7 (+), GCDFP-15 (–), ER (–), PR (–), Villin (–), CDX-2 (–), and CK20 (–) (**Figure 3**). In conjunction with the patient’s history of breast cancer and the immunophenotypic profile, a final diagnosis of small intestinal metastasis from breast cancer was established. The patient subsequently elected to receive further treatment at another institution.

**Figure 1:**
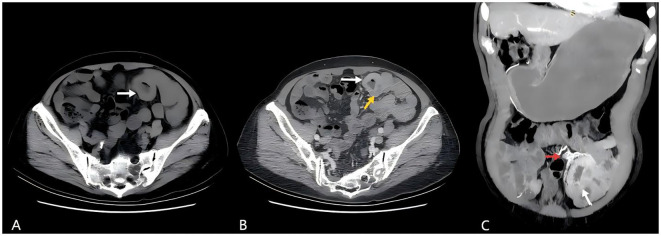
Contrast−enhanced CT revealed segmental, asymmetric circumferential thickening of the small bowel wall involving the third and fourth groups (corresponding to the jejunum and portions of the ileum). **(A)** (non−contrast CT) shows bowel wall thickening with attenuation values ranging from approximately 22 to 57 HU. **(B)** demonstrates heterogeneous enhancement of the thickened bowel wall after intravenous contrast administration, manifested as focal pronounced homogeneous enhancement (white arrows) and focal mild−to−moderate heterogeneous enhancement (yellow arrows). **(C)** shows bowel wall thickening with increased attenuation on delayed−phase imaging, accompanied by multiple enlarged perienteric lymph nodes (red arrows).

**Figure 2:**
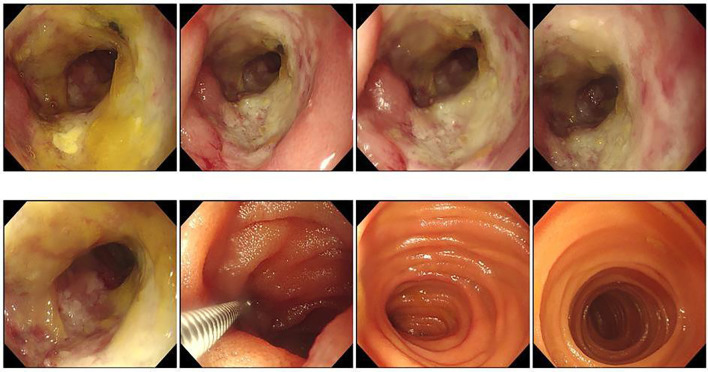
Enteroscopic view of the fourth segment of the small bowel showing a circumferential deep ulcerative lesion measuring 5–6 cm in length, covered with yellowish-white exudate, with firm consistency, surrounding mucosal erosion and edema. The lesion was friable with easy post-biopsy bleeding.

**Figure 3:**
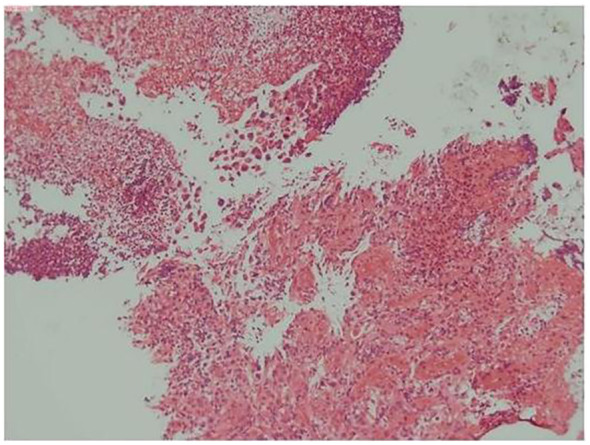
Pathological diagnosis of the small bowel biopsy (site 1): poorly differentiated adenocarcinoma with necrosis. Immunohistochemical staining revealed GATA-3 (+), CK7 (+), GCDFP (–), ER (–), PR (–), Villin (–), CDX-2 (–), and CK20 (–). Correlating with the clinical history and immunohistochemical profile, these findings were consistent with metastatic breast carcinoma. Biopsy from site 2 showed chronic mucosal inflammation.

## Discussion

Breast cancer is among the most common malignancies in women. In 2020, it accounted for approximately 11.7% of all newly diagnosed cancer cases worldwide, ranking it as one of the leading global malignancies (1). TNBC constitutes 15–20% of all breast cancer cases and is characterised by high aggressiveness and a relatively elevated risk of recurrence and metastasis (2). The most frequent sites of distant metastasis from breast cancer are the bone, lung, liver, and central nervous system; gastrointestinal involvement is comparatively rare, and small intestinal metastasis is particularly uncommon (3). The rarity of small intestinal metastasis from breast cancer may be attributable to the unique physiological and immune microenvironment of the small intestine.

The clinical manifestations of small intestinal metastasis from breast cancer are diverse, with intestinal obstruction-related abdominal pain being the most common initial symptom. In the present case, however, anaemia was the sole presenting feature. We attribute this to three contributing factors: (i) taxane-based chemotherapy-induced myelosuppression, which impairs haematopoiesis; (ii) chemotherapy-induced mucosal injury of the small intestine, leading to malabsorption of iron and vitamin B_12_; and (iii) metastatic infiltration along the bowel wall without luminal stenosis or overt ulceration, thereby accounting for the absence of abdominal pain or obstructive symptoms, yet the lesions persistently disrupt the mucosal microvasculature, resulting in occult chronic blood loss. Prolonged iron depletion ultimately precipitates iron-deficiency anaemia, which explains the exclusive initial presentation with anaemia (4–6).

The precise mechanisms underlying small intestinal metastasis from breast cancer remain to be fully elucidated. Current evidence suggests that haematogenous spread is the predominant route, followed by lymphatic dissemination, whereas peritoneal seeding is relatively uncommon (3). The gastrointestinal metastasis rate is 1.1% for invasive ductal carcinoma and 4.5% for invasive lobular carcinoma (7, 8). Immunophenotypically, oestrogen receptor (ER)- and progesterone receptor (PR)-positive breast cancers exhibit a higher propensity for gastrointestinal and peritoneal metastases, whereas the metastatic rate in triple-negative breast cancer (TNBC) is less than 1% (7). In contrast to invasive lobular carcinoma (ILC), which is characterised by complete loss of E-cadherin, invasive ductal carcinoma (IDC) displays downregulation of E-cadherin—a key mechanism underlying its metastatic potential (3). The small intestine is among the most highly vascularised digestive organs, possessing a dense submucosal capillary and venular network that facilitates the arrest, adhesion, and transvascular passage of circulating tumour cells. Reduced E-cadherin expression diminishes intercellular adhesion, allowing individual tumour cells or small clusters to detach from the primary lesion (9). Consequently, IDC cells tend to disseminate in cohesive clusters, readily forming microemboli within submucosal vessels, from which they invade the deeper intestinal wall layers, including the muscularis propria and serosa. Under microenvironmental stimuli such as hypoxia and tumour necrosis factor-α (TNF-α), these cells undergo epithelial–mesenchymal transition (EMT), accompanied by activation of transcription factors including Slug, Snail, and Twist, which enhance migratory and invasive capacities, facilitating transvascular and translymphatic spread to distant sites. Concurrently, mutational activation of the PIK3CA/AKT/mTOR and RAS/RAF/MEK pathways promotes proliferation and suppresses apoptosis, enabling tumour cells lodged in the intestinal wall to evade immune-mediated clearance. Thus, owing to the relative preservation of E-cadherin expression and consequently greater intercellular adhesion compared with ILC, IDC exhibits a lower incidence of small intestinal metastasis; the resulting lesions are typically nodular in morphology—a feature that explains the bowel wall thickening observed on imaging (10–12).

Compared with previously reported cases, the primary pathology in the present case was invasive ductal carcinoma of triple-negative phenotype, and small intestinal metastasis was relatively uncommon, rendering this case distinct in terms of pathological type, molecular subtype, and initial clinical presentation (13). Thus, it merits documentation as a clinical report. Furthermore, the interval between the diagnosis of primary breast cancer and the detection of intestinal metastasis is often long, typically spanning several years. Some studies report a median interval of approximately 7–10 years, whereas others describe a mean interval of about 4 years, with the longest documented interval reaching 28 years (7, 14). Owing to the prolonged latency period and the non-specific clinical manifestations, such cases are subject to misdiagnosis as Crohn’s disease, primary small intestinal tumours, or other inflammatory conditions in clinical practice (15).

Breast cancer metastases to the small intestine predominantly involve the serosal, muscular, and submucosal layers. Tumour cells display a single-file arrangement and infiltrate along stromal spaces (11), with abundant fibrous tissue proliferation within the stroma, while the mucosal surface may remain relatively intact. Consequently, endoscopy alone may have limited sensitivity for timely lesion detection or acquisition of representative pathological tissue, necessitating adjunctive imaging studies for diagnostic evaluation. As the core imaging modality, computed tomography (CT) can provide valuable diagnostic clues. Based on previous literature and the CT findings in the present case, the degree of bowel wall thickening is categorised as mild (3–5 mm), moderate (5–9 mm), or severe (≥10 mm) (16). We preliminarily summarise the imaging features as segmental, asymmetric small bowel wall thickening with adjacent lymphadenopathy. Owing to abundant fibrous tissue proliferation within the tumour stroma, luminal narrowing is absent. The thickened wall exhibits heterogeneous enhancement, which may reflect variations in tumour-derived vasculature. Specifically, areas of mild thickening show mild homogeneous enhancement, areas of moderate thickening demonstrate marked homogeneous enhancement, whereas areas of severe thickening display mild-to-moderate heterogeneous enhancement, potentially attributable to central ischaemia within the tumour (17). Small intestinal lymphoma commonly arises in the terminal ileum, where lymphoid tissue is abundant. Imaging findings include segmental, diffuse, moderate-to-marked symmetric bowel wall thickening. Lymphoma is hypovascular, exhibiting mild homogeneous enhancement and typically involving a longer segment, without luminal narrowing. Perienteric lymphadenopathy may be present, and adjacent fat planes are preserved (18). Primary small intestinal adenocarcinoma most frequently occurs in the duodenum. In early stages, the bowel wall shows relatively uniform thickening with homogeneous moderate enhancement; in intermediate and advanced stages, asymmetric wall thickening is often observed, with luminal stenosis occurring earlier and more severely. Lymph node metastasis tends to occur earlier, while hepatic metastasis is more common in advanced stages (19). Crohn’s disease is a chronic granulomatous disorder that may affect any segment of the gastrointestinal tract from the mouth to the anus; the small intestine—particularly the terminal ileum—is the most common site of involvement. Typical imaging features include segmental bowel wall thickening and mild luminal stenosis, which usually does not cause obstruction. The degree of wall thickening generally does not exceed 15 mm, and surrounding exudative changes may be present. On contrast-enhanced imaging, it shows segmental enhancement, with a “comb sign” in the adjacent mesentery and marked enhancement of the mesenteric border of the bowel wall. Enlarged lymph nodes are rarely involved (20, 21).

Beyond the distinct imaging features, definitive differentiation between primary gastrointestinal neoplasms and breast cancer metastases relies on histopathological and immunohistochemical evaluation. GATA-3 positivity is the core evidence of mammary origin. The immunophenotype of mammary metastases is characteristically defined by co-expression of mammaglobin and cytokeratin 7 (CK7), along with negativity for cytokeratin 20 (CK20)—a key combinatorial panel that distinguishes metastatic breast lesions from primary intestinal tumours (3).

## Conclusion

Breast cancer metastasis to the small intestine is extremely rare and is often difficult to detect or misdiagnosed in clinical practice. For patients with a history of breast cancer presenting with unexplained anemia, positive faecal occult blood, or abnormal gastrointestinal imaging findings (even in the absence of obvious gastrointestinal symptoms), the possibility of small intestinal metastasis should be considered. Imaging findings include segmental asymmetric thickening of the small bowel wall, extensive fibrous tissue proliferation within the tumour stroma, absence of luminal stenosis, and heterogeneous enhancement of the thickened bowel wall. These features may help differentiate this condition from other intestinal diseases. A comprehensive analysis integrating the patient’s medical history, endoscopic biopsy, pathology and immunohistochemistry is helpful for early diagnosis and guiding subsequent treatment.

## Data Availability

The original contributions presented in the study are included in the article/supplementary material. Further inquiries can be directed to the corresponding authors.
